# Can foot anthropometric measurements predict dynamic plantar surface contact area?

**DOI:** 10.1186/1757-1146-2-28

**Published:** 2009-10-28

**Authors:** Thomas G McPoil, Bill Vicenzino, Mark W Cornwall, Natalie Collins

**Affiliations:** 1 The Laboratory for Foot & Ankle Research, Department of Physical Therapy and Athletic Training, Northern Arizona University, Flagstaff, AZ, 86011, USA; 2 Division of Physiotherapy, School of Health and Rehabilitation Sciences, University of Queensland, St. Lucia, Queensland, Australia

## Abstract

**Background:**

Previous studies have suggested that increased plantar surface area, associated with pes planus, is a risk factor for the development of lower extremity overuse injuries. The intent of this study was to determine if a single or combination of foot anthropometric measures could be used to predict plantar surface area.

**Methods:**

Six foot measurements were collected on 155 subjects (97 females, 58 males, mean age 24.5 ± 3.5 years). The measurements as well as one ratio were entered into a stepwise regression analysis to determine the optimal set of measurements associated with total plantar contact area either including or excluding the toe region. The predicted values were used to calculate plantar surface area and were compared to the actual values obtained dynamically using a pressure sensor platform.

**Results:**

A three variable model was found to describe the relationship between the foot measures/ratio and total plantar contact area (*R*^2 ^= 0.77, *p *< 0.0001)). A three variable model was also found to describe the relationship between the foot measures/ratio and plantar contact area minus the toe region (*R*^2 ^= 0.76, *p *< 0.0001).

**Conclusion:**

The results of this study indicate that the clinician can use a combination of simple, reliable, and time efficient foot anthropometric measurements to explain over 75% of the plantar surface contact area, either including or excluding the toe region.

## Background

In attempting to understand a patient's foot morphology, the clinician should not only evaluate foot posture and mobility but should also consider assessing the amount of plantar surface contact area. The need for the clinician to assess the amount of plantar surface area would appear to be justified since previous investigators have reported that increased plantar surface contact area, associated with pes planus, can be a risk factor in the development of overuse injuries[1,2].

Two of the three studies that have attempted assess the relationship between plantar surface area and overuse injuries of the lower extremity have reported that an increase in plantar surface area would appear to be a risk factor. While Michelson et al[3] found that increased plantar surface area was not a risk factor for lower extremity injuries in an athletic population, both Kaufman et al[1] and Levy et al[2] reported that increased plantar surface area associated with a pes planus foot type caused an increased level of lower extremity overuse injuries in military populations. In the most recent study by Levy et al, plantar surface area was assessed on 512 West Point cadets, within one week of enrollment[2]. The cadets were then followed for 46 months as they underwent a prescribed amount of high-level physical activity. These authors used specific guidelines to define those individuals with pes planus based on footprints obtained using a Harris and Beath mat. They reported that over the four-year period of the study, those cadets with an increased plantar surface area had a significantly greater number of lower extremity overuse injuries.

While the need for the clinician to assess plantar surface contact area would appear to be indicated, the methods used for obtaining plantar surface area impressions range from the use of footprints obtained from an inked mat to more sophisticated sensor platforms used to measure plantar pressures as well as surface area. While an inked mat system is economical, obtaining footprints as well as the analysis is time intensive. While state-of-the-art pressure sensor platform systems allow the clinician to quickly obtain and analyze footprints, the necessary equipment and software is very costly. Ideally, it would be most advantageous for the clinician to be able to determine a patient's plantar surface area using simple, reliable, and time efficient anthropometric measurements of the foot.

Interestingly, previous studies have attempted to use plantar surface area in an attempt to predict foot posture, in particular, the height of the medial longitudinal arch. Unfortunately, none of these studies have been able to explain more than 55% of the bony height of the medial longitudinal arch using the amount of plantar surface area in contact with the ground[4-7]. To date no study has attempted to use foot anthropometric measurements to predict plantar surface contact area, even though increased plantar surface area associated with pes planus has been identified as a possible risk factor in the development of lower extremity overuse injuries. Thus, the purpose of this study was to determine if the use of a single or combination of simple, reliable, and time efficient foot anthropometric measurements could be used to predict plantar surface contact area.

## Methods

### Participant characteristics

One hundred and fifty-five individuals (97 females and 58 males) volunteered to participate in the study. Participants were recruited from the Northern Arizona University population and the surrounding Flagstaff, Arizona community. All participants met the following inclusion criteria: 1) no history of congenital deformity in the lower extremity or foot; 2) no previous history of lower extremity or foot fractures; 3) no systemic diseases that could affect lower extremity or foot posture; and 4) no history of trauma or pain to either foot, lower extremity, or lumbosacral region at least 12 months prior to the start of the investigation. Volunteers with any visual signs of hallux valgus or other toe deformities were also excluded from participation. The mean age of the 155 participants was 24.5 ± 3.5 years with a range of 18 to 39 years. The Institutional Review Board of Northern Arizona University (IRB # 07.0233) approved the protocol for data collection and all participants provided written informed consent prior to participation.

### Instrumentation

**Figure 1:**
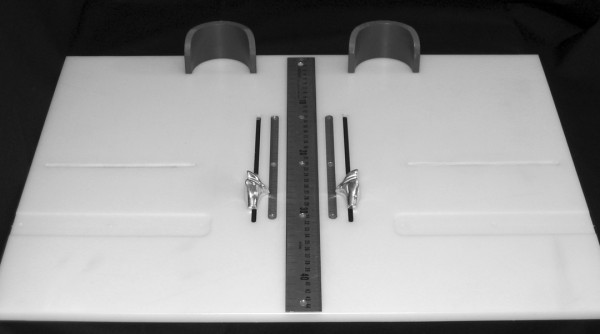
**Foot Measurement Platform**.

**Figure 2:**
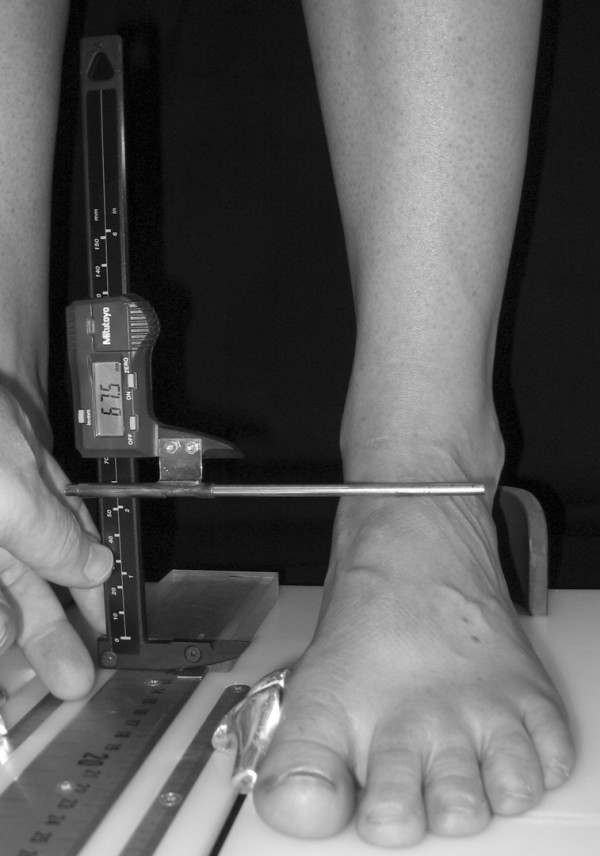
**Digital gauge used to measure dorsal arch height**.

**Figure 3:**
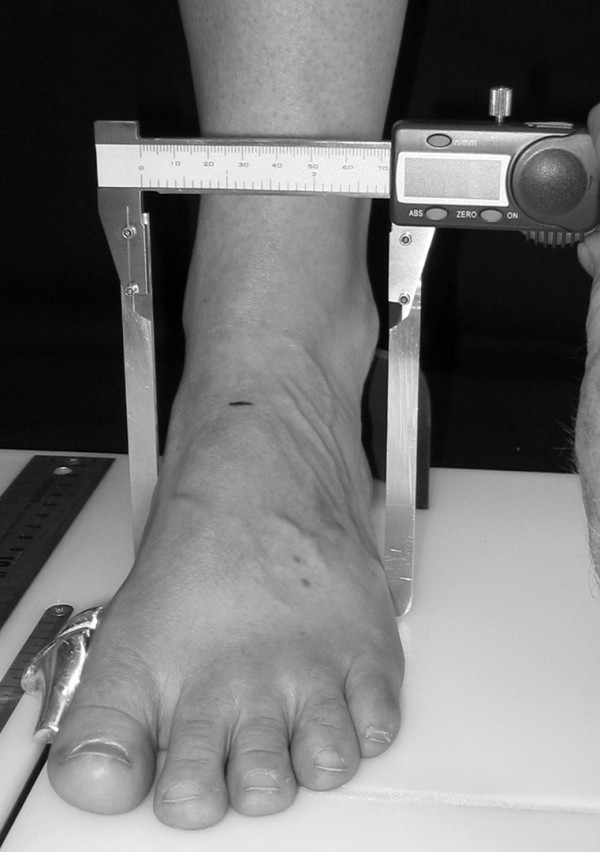
**Digital caliper for measuring foot widths being used to measure midfoot width**.

To obtain the foot anthropometirc measurements, a foot measurement platform that has been previously described was utilized (Figure 1) [8]. In addition to the foot measurement platform, two instruments were manufactured for the study to permit the measurement of both arch height and the various foot widths. The weight bearing arch height gauge consisted of a digital caliper (Model #700-126, Mitutoyo USA, Aurora, IL 60502) with the fixed point attached to a 1.2 × 5.0 × 10.0 cm plastic block to hold the caliper in a vertical position and a sliding metal rod attached to the moving point of the caliper to permit the assessment of arch height (Figure 2). A second digital caliper (Model # S54-101-150-2, Fowler Equipment, Newton, MA 02466) was modified, to permit the measurement of forefoot, midfoot, and heel widths by attaching 0.03 × 0.8 × 9.0 cm metal plates to both the fixed and the moving points of the caliper (Figure 3).

To obtain dynamic plantar surface contact area during walking, an EMED-X floor mounted capacitance transducer platform (NOVEL USA, Inc, Minneapolis, MN, 55415), with an active sensor area of 32 × 47.5 cm, was positioned at the midpoint of a 12-meter walkway to collect dynamic plantar surface area during walking. The EMED-X platform had a matrix of 6080 sensors with a density of four sensors per cm^2 ^and a sampling rate of 100 Hz. The input pressure saturation range for the capacitance sensors used in the EMED platform is 1270 kPa. In the current study, none of the trials performed by any subject achieved pressure saturation levels for the platform system.

### Procedures

**Figure 4:**
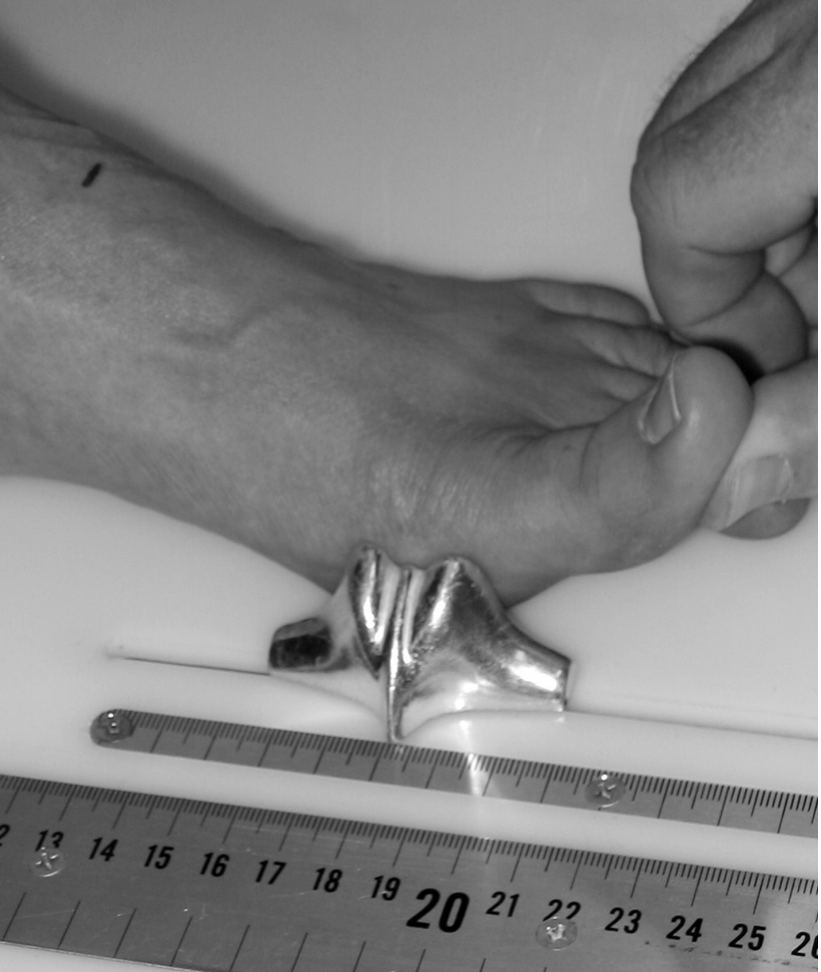
**Extending first metatarsophalangeal joint to ensure proper placement of indicator**.

**Figure 5:**
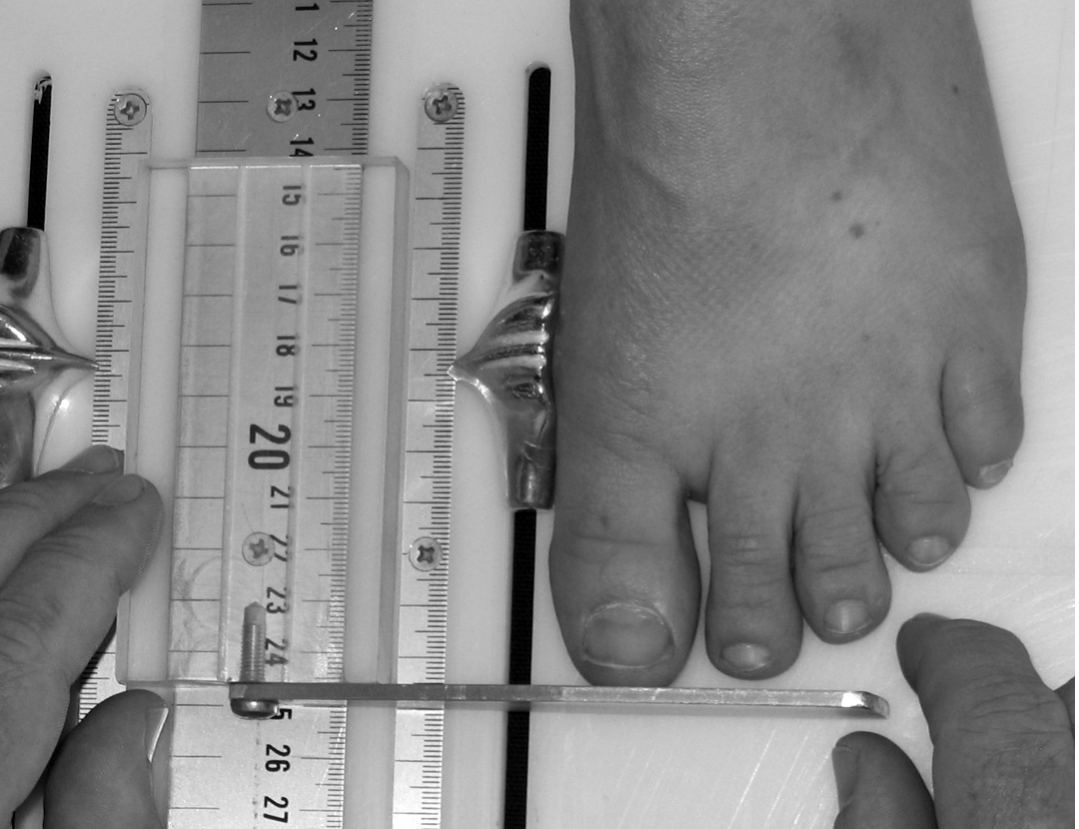
**Placement of forefoot using first metatarsophalangeal joint indicator and measurement of total foot length**.

**Figure 6:**
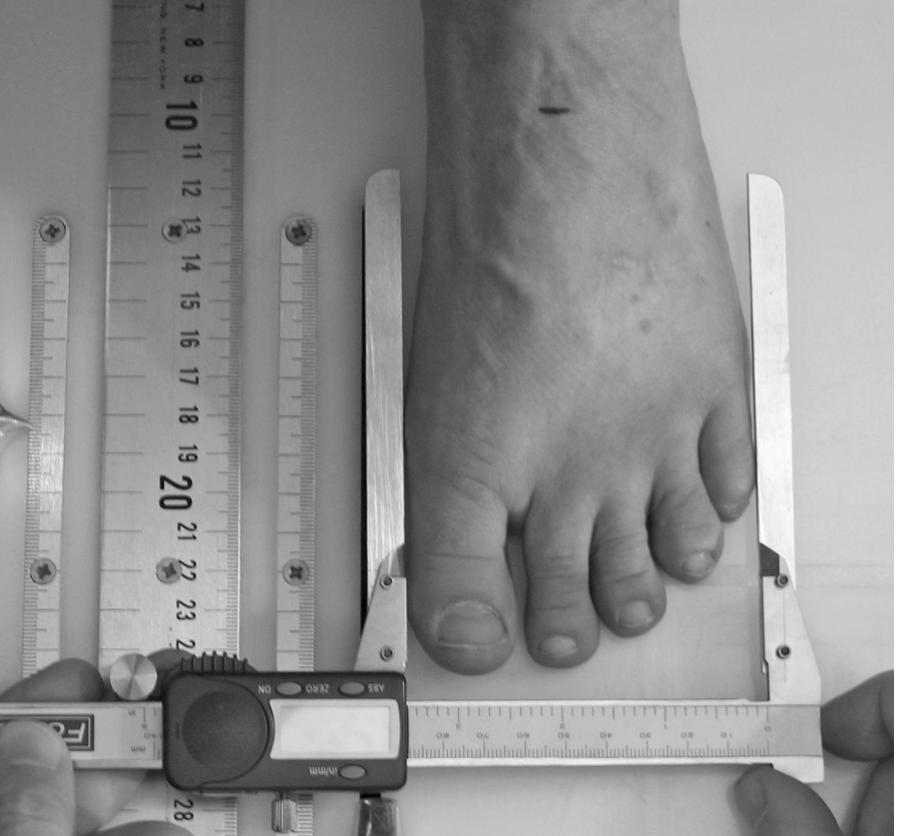
**Measurement of forefoot width**.

**Figure 7:**
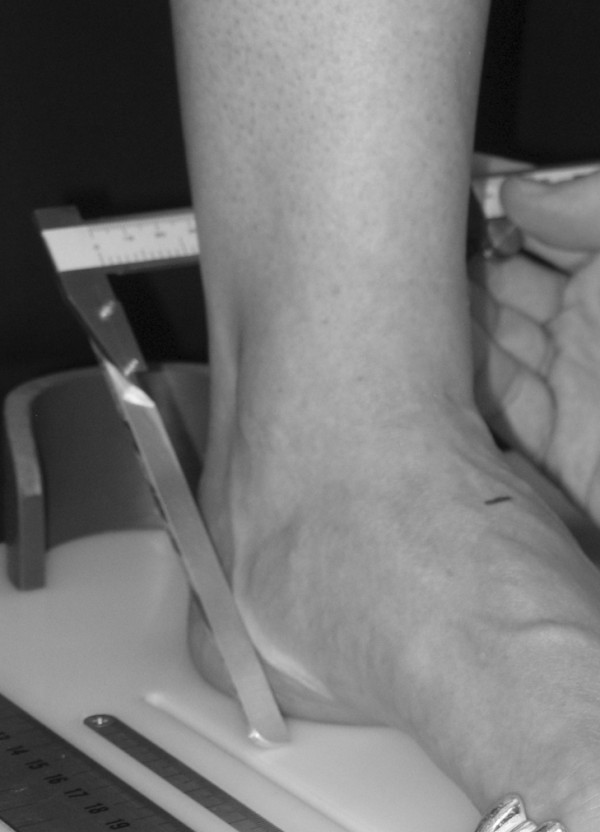
**Placement of the digital caliper at 45° to the platform to measure heel width**.

**Figure 8:**
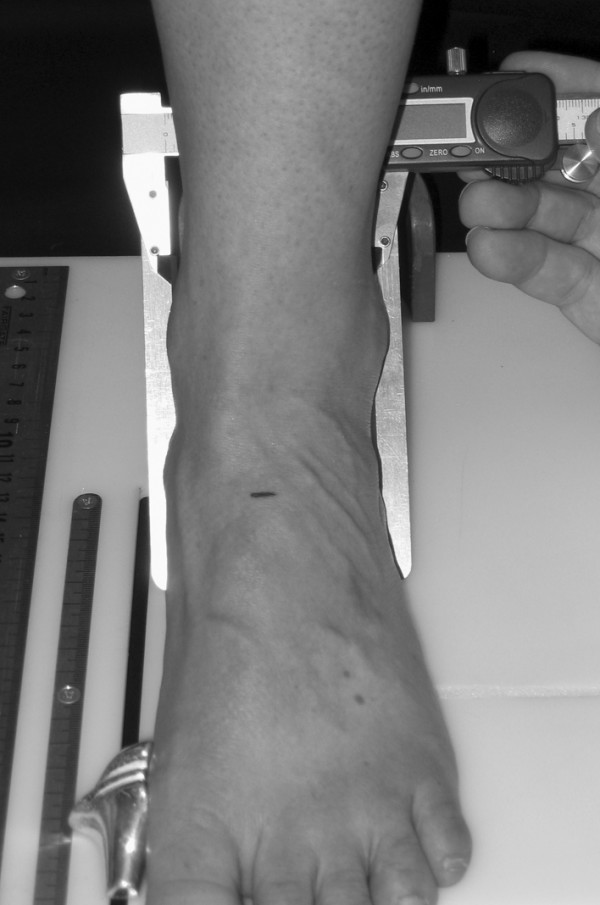
**Measurement of heel width**.

After height and weight were obtained, each subject was asked to stand on the foot measurement platform with both heels placed in left and right heel cups that were positioned 15.24 cm apart. Next, the sliding first metatarsophalangeal joint indicator was positioned over the medial prominence of the first metatarsal head. To ensure the proper placement of the indicator over the medial prominence of the first metatarsal head, the examiner ensured that the hallux could be extended without causing any displacement of the indicator (Figure 4). Once the first metatarsophalangeal joint indicator was properly positioned bilaterally, the subject was instructed to place equal weight on both feet so that the following weight-bearing measurements could be obtained. Total foot length was first measured by placing the sliding bar on the centered metal ruler attached to the platform and moving the bar to just touch the longest toe, usually the hallux, of each foot (see Figure 5). Ball length (BL) was recorded based on the position of the first metatarsophalangeal joint indicator in relation to offset metal rulers that were aligned with the centered metal ruler (see Figure 5). Total foot length was divided in half and the dorsums of both feet were marked at the 50% length point using a water-soluble pen. The sliding metal rod of the weight bearing height gauge was then positioned over the 50% length mark and the vertical height from the top of the platform to the dorsum of each foot (DAH) was measured (see Figure 2). Next, the caliper designed to assess foot width was used to measure forefoot width (FFWid) by positioning the edges of the two metal arms attached to the caliper so that they were parallel to the centered metal ruler on the platform (Figure 6). The metal arms were then moved until they just made contact with the skin. Once both rods made contact with the skin, the FFWid was recorded. To assess midfoot width (MFWid), the digital caliper was positioned so that the arms of the caliper were aligned laterally and medially to the 50% length point marked on the dorsum of the foot (see Figure 2). The lateral and medial arms where then moved until they just made contact with the skin at the 50% length point. The MFWid was then recorded. To assess heel width (HLWid), the subject was asked to slide both feet forward on the measurement platform, so both heels were no longer in the heel cups. Care was taken to ensure that the subject did not change the alignment of their feet as they slide their feet forward on the platform. The digital width caliper was then placed behind each heel with the metal arms of the caliper placed at a 45° angle to the platform (Figure 7). The arms were then moved together until they just made contact with the skin on the lateral and medial sides of the heel (Figure 8). Once both rods made contact with the skin, the HLWid was recorded. The use of 50% of the total foot length for both the dorsal arch height and the midfoot width measurements was based on the results of previous research assessing the consistency for the measures of arch height and midfoot width [8,9]. All measurements were obtained on both feet of all 155 subjects by the same rater and were recorded in centimeters. The maximum time required for completing all six measurements on both feet ranged from 5 to 6 minutes.

Once the foot measurements were completed, each subject was then instructed to practice walking barefoot at a self-selected speed along the 12-meter walkway for several minutes. In order to prevent targeting of the EMED-X platform, subjects were instructed not to look at the ground while walking. Walking speed was monitored using a digital stopwatch to time the subject as they walked between two lines positioned 6.1 meters apart and were equidistant in relation to the platform. When between-trial walking speed was consistent (variation of less than 5 percent between trials), each subject was asked to walk barefoot over the walkway while data were recorded from the EMED-X platform for five trials on both the left and right foot.

### Determination of Reliability

To assess the reliability for the six (6) foot measurements, three raters were asked to assess the left and right feet of 12 randomly selected participants. The raters performing the measurements were three physical therapists with a minimum of 2 years clinical experience (mean experience 16 years; range 2 to 30 years). Each rater attended a single one-hour training session to receive verbal instructions as well as to practice the techniques to ensure that they were taking the measurements correctly. The reliability data collection consisted of two sessions, one-week apart, in which each rater performed all six measurements on both feet of all 12 subjects. Each rater was blinded from all measurements and the mark placed over the dorsum of each foot was removed after each set of measurements to prevent subsequent rater bias. The left and right feet for all 12 subjects were treated as independent observations so that the analysis of reliability was conducted on 24 feet.

### Data Analysis

In addition to the six (6) foot measurements (TFL, BL, DAH, FFWid, MFWid, HLWid), the arch height ratio (AHRatio) was also calculated. To determine the AHRatio, the DAH was divided by the BL. To determine plantar surface contact area, a standardized four region masking model (Novel Automask, NOVEL USA, Inc, Minneapolis, MN, 55415) was used to divide the dynamic plantar surface area into four regions; rearfoot, midfoot, forefoot, and toes. The heel to midfoot and midfoot to forefoot regions were defined by using 73% and 45% of the entire foot length from the toes to the heel, respectively. The forefoot to toe region was defined by using the pressure gradients around the peak pressures of the toes. The Novel Groupmask program (NOVEL USA, Inc, Minneapolis, MN, 55415) was used to determine the mean plantar surface contact area for the five trials collected on each subject's feet. Two different plantar surface contact areas were calculated; 1) the total plantar surface contact area (TPCA), which included all four plantar regions, and 2) plantar surface contact area minus the toe region (PCA-Toes).

### Statistical Analysis

Intraclass correlation coefficients (ICC) were calculated to determine the consistency of each rater to perform the measurements repeatedly both individually (intra-rater; ICC_3,1_) as well as in comparison to the other raters (inter-rater; ICC_2,3_)[10]. The level of reliability for the ICC was classified using the characterizations reported by Landis and Koch[11]. These characterizations were: *slight*, if the correlation ranged from .00 to .21; *fair*, if the correlation ranged from .21 to .40; *moderate*, if the correlation ranged from .41 to .60; *substantial*, is the correlation ranged from .61 to .80; and *almost perfect*, if the correlation ranged from .81 to 1.00. Although the ICC is a well accepted measure of reliability, it is difficult to interpret ICC values since they are dependent on the variability of the group being assessed and may not transfer to different patient populations [12]. Thus in addition to ICC values, the standard error of the measurement (SEM) was also calculated as another index of reliability. The SEM is a number in the same units as the original measurement that represents the way a single score would vary if the six foot measurements used in this study were measured more than once [13]. In addition to descriptive statistics, *t*-tests were used to determine whether extremity differences for females and males existed for the seven anthropometric measures of the foot.

The six (6) foot measures and one (1) ratio were entered into a stepwise forward linear regression to determine the most parsimonious set of variables associated with TPCA and PCA-Toes. A significance level of p < 0.05 was required for entry into the model and p < 0.06 was used as the criteria for removal from the model. Using the regression equation that was developed, predicted mean values for TPCA and PCA-Toes were calculated and compared using t-tests to the measured mean values for TPCA and PCA-Toes mean values. All statistical analyses were performed using JMP software, Version 8.0 (SAS Institute Inc, Cary, NC 27513). An alpha level of .05 was established for all tests of significance.

## Results

Demographic data for all 155 subjects are listed in Table 1. The intra-rater and inter-rater ICC and SEM values are shown in Tables 2 and 3. The intra-rater reliability for all six (6) foot measurements ranged from 0.98 to 0.99 for all three raters regardless of experience level. The intra-rater SEM values ranged from 0.02 to 0.08 centimeters and were all less than 2% of the actual measurement value. The inter-rater reliability ICC for the same measurements ranged from 0.98 to 0.99 for both day one and day two with SEM values ranging from 0.03 to 0.10 centimeters.

**Table 1:** Subject characteristics with values presented as mean, standard deviation (SD), and 95% confidence Intervals (CI).

N = 155	MEAN	SD	95% CI
**Age (years)**	24.6	3.5	24.1 - 25.2
**Height (cm)**	171.0	9.0	169.6 - 172.4
**Weight (kg)**	71.2	14.0	68.9 - 73.5
**Body Mass Index (kg/m2)**	24.3	3.9	23.6 - 24.9

**Table 2:** Intra-rater reliability coefficients (ICC) and standard error of the measurement (SEM)

	Rater 1 (30 years experience)			Rater 2 (16 years experience)			Rater 3 (2 years experience)		
	**ICC**	**Mean (cm)**	**SEM (cm)**	**ICC**	**Mean (cm)**	**SEM (cm)**	**ICC**	**Mean (cm)**	**SEM (cm)**

**Foot Length**	0.99	25.96	0.04	0.99	26.05	0.04	0.99	25.96	0.04
**Ball Length**	0.98	18.98	0.08	0.98	18.95	0.07	0.99	19.05	0.04
**Ball Width**	0.98	09.70	0.05	0.99	08.88	0.05	0.98	08.61	0.06
**Midfoot Width**	0.98	08.82	0.04	0.99	08.88	0.03	0.99	08.61	0.04
**Heel Width**	0.99	06.66	0.02	0.99	06.80	0.02	0.99	06.87	0.04
**Dorsal Arch Hgt**	0.98	06.52	0.03	0.98	06.53	0.03	0.98	06.52	0.03

**Table 3:** Inter-rater reliability coefficients (ICC) and standard error of the measurement (SEM) for day 1 and day 2.

	Day 1		Day 2	
	**ICC**	**SEM**	**ICC**	**SEM**

**Foot Length**	0.99	0.06	0.99	0.05
**Ball Length**	0.99	0.07	0.98	0.07
**Ball Width**	0.99	0.04	0.99	0.04
**Midfoot Width**	0.98	0.10	0.99	0.08
**Heel Width**	0.98	0.07	0.98	0.08
**Dorsal Arch Hgt**	0.98	0.04	0.98	0.03

Descriptive statistics for all measurements are listed by extremity and gender in Table 4. The results of the *t*-tests indicated that there were no significant differences between the left and right feet for any of the foot measurements for either the female or male subjects. In addition, a stepwise forward linear regression analysis was performed for both the left and right feet of all subjects to predict TPCA and PCA-Toes. For the left foot, the regression analysis resulted in a three variable (3) variable model (MFWid, HLWid, and AHRatio) for TCPA (R^2 ^= 0.79) and for PCA-Toes (R^2 ^= 0.78). For the right foot, the regression analysis resulted in the same three (3) variable model (MFWid, HLWid, and AHRatio) for TCPA (R^2 ^= 0.75) and for PCA-Toes (R^2 ^= 0.75). Based on the results of the t-tests and the fact that the regression prediction models determined for each foot were so similar, the left and right feet were grouped for the final regression analysis that is reported in this paper.

**Table 4:** Mean and standard deviations (SD) for the six foot measurements and the one ratio by gender and extremity.

	Foot Length	Ball Length	Ball Width	MIdfoot Width	Heel Width	Dorsal Arch Hgt	AHR
**FEMALES** **(N = 97)**							
**Left**	24.4 (1.2)	17.8 (0.9)	9.1 (0.5)	8.0 (0.6)	6.1 (0.4)	6.2 (0.4)	0.349 (0.026)
**Right**	24.4 (1.2)	17.9 (0.9)	9.2 (0.5)	8.1 (0.6)	6.1 (0.4)	6.1 (0.4)	0.342 (0.030)

**MALES** **(N = 58)**							
**Left**	26.7 (1.3)	19.5 (0.9)	10.2 (0.5)	9.1 (0.6)	6.8 (0.4)	6.9 (0.5)	0.354 (0.03)
**Right**	26.7 (1.2)	19.6 (0.9)	10.2 (0.6)	9.2 (0.6)	6.8 (0.4)	6.8 (0.5)	0.348 (0.030)

The stepwise forward linear regression analysis to predict TPCA for all 310 feet resulted in a three (3) variable model (F = 349.9, *p *< 0.0001) that had an *R*^2 ^= 0.77, and an adjusted *R*^2 ^= 0.77. The three measurements that were included in the model were: MFWid, HLWid, and AHRatio. The mean TPCA manually measured using the sensor platform was 116.9 cm^2 ^and the predicted TPCA based on the four measurements identified in the regression analysis was 116.9 cm^2^. The difference between the measured and predicted values for TPCA was 0.00 with a standard error of 0.55 cm^2^. A t-test indicated that there was no significant difference between the measured and predicted mean values for TPCA.

The stepwise forward linear regression analysis to predict PCA-Toes for all 310 feet also resulted in a three (3) variable model (F = 324.9, *p *< 0.0001) that had an *R*^2 ^= 0.76, and an adjusted *R*^2 ^= 0.76. The three measurements that were included in the model were the same as reported for TPCA: MFWid, HLWid, and AHRatio. The mean PCA-Toes manually measured using the sensor platform was 96.3 cm^2 ^and the predicted PCA-Toes based on the three measurements identified in the regression analysis was 96.3 cm^2^. The difference between the measured and predicted values for PCA-Toes was 0.00 with a standard error of 0.52 cm^2^. A t-test indicated that there was no significant difference between the measured and predicted mean values for PCA-Toes.

## Discussion

As previously noted, increased plantar surface area associated with pes planus would appear to be a possible risk factor in the development of lower extremity overuse injuries. While previous studies have attempted to predict bony arch height of the medial aspect of the foot using plantar surface contact, no investigations to date have attempted to use a single or combination of foot anthropometric measurements to predict plantar surface contact area. The intent of this study was to determine if a single or combination of foot anthropometric measurements could be used to predict plantar surface contact area.

The first issue in interpreting the results was the intra- and inter-rater reliability of the foot anthropometric measurements used in this study. The ICC values for all three raters, regardless of the number of years of clinical experience, would be classified as "almost perfect" for both intra-rater and inter-rater reliability based on the characterizations provided by Landis and Koch[11]. As noted in Tables 2 and 3, the SEM values were also quite small ranging from 0.02 to 0.10 cm for all six (6) foot measurements used in this study. Based on these findings, the authors concluded that the reliability of the measurement techniques used in the study was acceptable and that further analysis of the results could be performed.

*T*-tests indicated that significant differences did not exist between the left and right feet for both the female and male subjects. In addition, the results of linear regression analyses performed on both the left and right feet resulted in the same three variable model to predict TPCA and PCA-Toes. All of the prediction models determined for the left and right feet explained at least 75% of the plantar surface contact area either including or excluding the toe region. Based on the results of the *t*-tests and the fact that the regression prediction models for each foot were so similar, the left and right feet were grouped for the final regression analysis so that a single prediction model could be provided for TPCA as well as PCA-Toes based on all 310 feet.

Based on the results of the regression analysis on all 310 feet, the use of MFWid, HLWid, and AHRatio can explain more than 75% of the variance of TPCA. The small standard error of the mean (0.55 cm^2^) between the predicted and actual values for TCPA indicates the relative strength and utility of the resulting regression equation for the clinician to predict TPCA. This finding is further substantiated by the non-significant *t*-tests between the predicted and actual values. Using data from the current study, the clinician can predict the TPCA based on the selected foot measurements using the following formula:

Based on the results of the regression analysis on all 310 feet, the use of MFWid, HLWid, and AHRatio, also explains over 75% of the variance of PCA-Toes. The small standard error of the mean (0.52 cm^2^) between the predicted and actual values for PCA-Toes indicates the relative strength and utility of the resulting regression equation for the clinician to predict PCA-Toes. This finding is further substantiated by the non-significant *t*-tests between the predicted and actual values. Using data from the current study, the clinician can predict the PCA-Toes based on the selected foot measurements using the following formula:

The findings of this study indicate that the clinician can use a combination of simple, reliable, and time efficient foot anthropometric measurements/ratios to explain over 75% of the plantar surface contact area either including or excluding the toe region and to accurately predict an individual's plantar surface contact area. While prediction equations have been provided for both TPCA and PCA-toes, the choice of whether to use TPCA or PCA-Toes would be determined by clinician preference. While TPCA provides the clinician information on the total amount of surface area in contact with the supporting surface including the toes, in those cases where the client or patient had digital deformities such as hallux valgus, bunionette, claw or hammer toes, the use of PCA-Toes may be more preferable.

While the regression models described in this paper can explain more than 75% of TPCA and PCA-Toes when using a combination of foot measurements/ratios, previous attempts to use plantar surface contact area to predict bony arch height have been less successful. While the findings reported by Chu et al[5] and Shiang et al[6] indicated that approximately 50% of the bony height of the medial longitudinal arch could be explained on the basis of plantar contact area, in contrast Hawes et al[7] and McPoil et al[8] found that plantar surface contact area could only explain approximately 4% to 27% of the bony height of the medial longitudinal arch. Based on the results of the current study, it would appear that the use of foot measurements provides the clinician with a much more robust prediction of plantar surface contact area in comparison to using plantar surface area in an attempt to predict foot structure such as the bony height of the medial longitudinal arch. Conceptually, it would appear that the incorporation of the foot width measurements had the greatest influence on the regression model to predict plantar surface area. For both TPCA and PCA-Toes, midfoot width was the variable that had the greatest effect on the fit of the model.

In the current study, plantar contact surface area was recorded while participants walked across a pressure sensor platform. Urry and Wearing have reported that footprints obtained from certain types of pressure platforms with decreased sensor resolution are not the same as footprints obtained using an inked mat when the data is collected during static standing[14]. Although the pressure sensor platform used in the current study had a greater sensor resolution than the platform system that was used by Urry and Wearing in their study, care should be used when comparing the results reported in this study with measures of plantar surface area obtained using a inked mat in standing. The authors believe that obtaining data dynamically, in activities such as walking, provides a more functional representation of plantar surface contact area in comparison to static standing.

A limitation of the current study was the assumption by the authors that when subjects were asked to stand and place equal weight on each foot, that the subject was placing 50% of their body weight on each foot. While various methodologies could have been utilized to ensure that each subject was placing 50% of their body weight on each foot while the measurements in standing were being recorded, for example, having the subject stand with one foot on a scale, the methodology used in the current study can be easily replicated by clinicians. Tesser et al has previously reported that the amount of asymmetry in weight distribution between extremities in relaxed standing is 4% or less in healthy subjects[15]. Furthermore, while there could be slight variations in weight bearing symmetry between extremities when a subject is asked to stand with equal weight placed on both feet, the high level of repeatability of the foot anthropometric measurements utilized in this study that were assessed over multiple days would suggest that any degree of asymmetry is negligible.

## Conclusion

While further research is always warranted, the results of this study indicate that the clinician can use a combination of simple, reliable, and time efficient foot anthropometric measurements/ratios to explain over 75% of the plantar surface contact area. Prediction equations provided allow the practitioner to predict total plantar contact surface area as well as plantar surface area minus the toe region, based on clinical interest.

## Competing interests

The authors declare that they have no competing interests.

## Authors' contributions

TGM conceived the study, participated in the design of the study, carried out data collection and analysis. BV participated in the design of the study and carried out data collection. MWC participated in the design of the study and carried out data analyses. NC carried out data collection. 
